# Supplementary material for: Systematic review and meta-analysis of the effectiveness of pre-pregnancy care for women with diabetes for improving maternal and perinatal outcomes
Source: PLoS One. 2020 Aug 18;15(8):e0237571. doi: 10.1371/journal.pone.0237571 (PMC7433888; doi:10.1371/journal.pone.0237571)
Supplement: S1 File — (DOCX) [file pone.0237571.s002.docx]

**Search Strategy**

Electronic databases searched:

PubMed/MEDLINE (1946 to January 2019)

EMBASE (1947 to January 2019)

CINAHL (1981 to January 2019)

Cochrane Library (up to January 2019)

Web of Science (Core Collection) (1900 to January 2019)

PubMed/MEDLINE search:

1. Diabetes
2. Pregnancy in diabetics [MeSH]
3. 1 OR 2
4. PCC[MeSH]
5. preconception*
6. pregestation*
7. pre-gestation*
8. Prepregnancy
9. Pre-pregnancy
10. Periconception*
11. “Planning pregnancy”
12. “Pregnancy planning”
13. “Family planning service”
14. Family planning services [MeSH]
15. 4 OR 5 OR 6 OR 7 OR 8 OR 9 OR 10 OR 11 OR 12 OR 13 OR 14
16. 3 AND 15

EMBASE search:

1. ('pregnancy'/exp/mj AND [embase]/lim)
2. ('diabetes mellitus'/exp/mj AND [embase]/lim)
3. 1 AND 2
4. (preconception* OR 'pre conception*' OR pregestation* OR 'pre gestation*' OR 'pre pregnancy' OR prepregnancy OR periconception*) AND [embase]/lim)
5. ('family planning' AND [embase]/lim)
6. ('pregnancy planning' AND [embase]/lim)
7. ('prepregnancy care' AND [embase]/lim)
8. 4 OR 5 OR 6 OR 7
9. 3 AND 8

CINAHL search:

1. (MM "Pregnancy in Diabetes+")
2. (MM "Diabetes Mellitus+")
3. 1 OR 2
4. (MM "Prepregnancy Care")
5. preconception* OR pre-conception* OR pregestation* OR prepregnancy OR periconception* OR planning pregnancy OR pregnancy planning OR family planning service OR family planning services
6. 4 OR 5
7. 3 AND 6

Cochrane Library search:

'(diabetes AND pregnancy) AND (preconception* OR pre-conception* OR pregestation* OR prepregnancy OR periconception* OR “planning pregnancy” OR “pregnancy planning” OR “family planning service”) in Title Abstract Keyword'

Web of Science search:

(diabetes AND pregnancy) AND (preconception* OR pre-conception* OR pregestation* OR prepregnancy OR periconception* OR “planning pregnancy” OR “pregnancy planning” OR “family planning service”)
