# Supplementary material for: Systematic review and meta-analysis of the effectiveness of pre-pregnancy care for women with diabetes for improving maternal and perinatal outcomes
Source: PLoS One. 2020 Aug 18;15(8):e0237571. doi: 10.1371/journal.pone.0237571 (PMC7433888; doi:10.1371/journal.pone.0237571)
Supplement: S2 File — (DOCX) [file pone.0237571.s003.docx]

**Table of excluded studies**

| **Reason for exclusion** | **Study** | **NO** |
| --- | --- | --- |
| ( Did not meet the inclusion criteria) | Correa 2012^1^ |  |
| ( Did not meet the inclusion criteria) | Delgado 2001^2^ |  |
| (Review ) | Earle 2 017^3^ |  |
| The data not extractable | Glinianaia 2012^4^ |  |
| ( Did not meet the inclusion criteria) | Gutaj Paweł 2015^5^ |  |
| (Review) | Janz1 995^6^ |  |
| ( Did not meet the inclusion criteria) | Jouatte F 1999^7^ |  |
| (Review ) | Kelleher 1994^8^ |  |
| discussing uptake of PCC | Keely 2012^9^ |  |
| (Review ) | Kirkland 1999^10^ |  |
| (Review) | Kitzmiller 1996^11^ |  |
| (Review) | Lacam 2010^12^ |  |
| ( Did not meet the inclusion criteria) | Litwak 1992^13^ |  |
| Editorial | Lorber 1995^14^ |  |
| ( Did not meet the inclusion criteria) | [Madanat](https://www.ncbi.nlm.nih.gov/pubmed/?term=Madanat%20AY%5BAuthor%5D&cauthor=true&cauthor_uid=27186158) 2016^15^ |  |
| ( Did not meet the inclusion criteria) | Mansell 2009^16^ |  |
| ( Did not meet the inclusion criteria) | Matsuba 1990^17^ |  |
| ( Did not meet the inclusion criteria) | Matsuba 1997^18^ |  |
| (Review) | MD EK 2012^19^ |  |
| The data not extractable | Moloney 2011^20^ |  |
| ( Did not meet the inclusion criteria) | Norgaard 2017^21^ |  |
| (Review) | Posner 2006^22^ |  |
| (Review) | Posner 2008^23^ |  |
| ( Did not meet the inclusion criteria) | Schmiegelow 2014^24^ |  |
| (Did not meet the inclusion criteria). | Schneider 2011^25^ |  |
| ( Did not meet the inclusion criteria) | Schneider 2012^26^ |  |
| (Review ) | Schneiderman 2010^27^ |  |
| (Did not meet the inclusion criteria). | Schneid-Kofman 2005^28^ |  |
| ( Did not meet the inclusion criteria) | Schoch 2018^29^ |  |
| ( Did not meet the inclusion criteria) | Schoenaker 2015^30^ |  |
| ( Did not meet the inclusion criteria) | Schoenaker 2016^31^ |  |
| ( Did not meet the inclusion criteria) | Schoenaker 2018^32^ |  |
| ( Did not meet the inclusion criteria) | Scholing 2018^33^ |  |
| (Review) | Schrander 1999^34^ |  |
| Data not extractable | Triparhi 2010^35^ |  |
| Co-intervention | Yamamoto 2018^36^ |  |

**References**

1. Correa A, Gilboa SM, Botto LD, Moore CA, Hobbs CA, Cleves MA, et al. Lack of periconceptional vitamins or supplements that contain folic acid and diabetes mellitus-associated birth defects. Am J Obstet Gynecol. 2012;206**(3)**: 218.e1-13.
2. Delgado Del Rey M, Herranz L, Martin Vaquero P, Janez M, Juan Lozano Garcia J, Darias R, et al. [Effect of preconceptional metabolic control in the course of pregnancy in diabetic patients]. Med Clin (Barc). 2001;117**(2)**:45-8.
3. Earle S, Tariq A, Komaromy C, Lloyd CE, Karamat MA, Webb J, et al. PCCfor women with type 1 or type 2 diabetes mellitus: a mixed-methods study exploring uptake of preconception care. Health technology assessment (Winchester, England). 2017;21**(14):**1-130.
4. Glinianaia SV, Tennant PW, Bilous RW, Rankin J, Bell R. HbA(1c) and birthweight in women with pre-conception type 1 and type 2 diabetes: a population-based cohort study. Diabetologia. 2012;55*(12)*:3193-203.
5. Gutaj P, Zawiejska A, Brazert J, Wender-Ozegowska E. Association between preconceptional treatment with insulin pumps and improved metabolic status in early pregnancy in women with type 1 diabetes. Pol Arch Med Wewn. 2015;125**(5)**:329-36.
6. Janz NK, Herman WH, Becker MP, Charron-Prochownik D, Shayna VL, Lesnick TG, et al. Diabetes and pregnancy. Factors associated with seeking pre-conception care. Diabetes Care. 1995;18**(2)**:157-65.
7. Jouatte F, Aitken B, Dufour P, Valat AS, Vamberghe A, Cappoen JP, et al. [Diabetes before pregnancy, apropos of 143 cases]. Contraception, fertilite, sexualite (1992). 1999;27**(12)**:845-52.
8. Kelleher A. Careful planning ensures a happy outcome. Caring for pregnant women with diabetes. Professional nurse (London, England). 1994;10**(2)**:116-20.
9. Keely E. Preconception Care for Women with Type 1 and Type 2 Diabetes—The Same But Different. Canadian Journal of Diabetes. 2012 ;36(2):83–6. 10.1016/j.jcjd.2012.03.001.
10. Kirkland F. Preconceptual care for women with diabetes. Journal of Diabetes Nursing. 1999; 3**(4):**5.
11. Kitzmiller JL, Buchanan TA, Kjos S, Combs CA, Ratner RE. Pre-conception care of diabetes, congenital malformations, and spontaneous abortions. Diabetes Care. 1996;19**(5)**:514-41.
12. Lacam C NJ. Diabetes during pregnancy: diabetes care and its complications from the preconceptional period to post-partum. Journal De Gynecologie Obstetrique Et Biologie De La Reproduction. 2010;39**(2):**176-8.
13. Litwak LE, Mileo Vaglio R, Fried T, De Sancho H, Alvarez A, Althabe O, et al. [Intensified insulin therapy in the management of gestational diabetes]. Medicina. 1992;52**(6)**:523-33.
14. Madanat AY, Sheshah EA. PCC in Saudi women with diabetes mellitus. Journal of family & community medicine. 2016;23**(2)**:109-14.
15. Mansell A, Gouveia, C., Braggins, F., Claydon, A., Nobeebux, A., Joseph, T., ... & Finer, S. Early screening for gestational diabetes is essential to detect undiagnosed impaired glucose tolerance and Type 2 diabetes in a high risk, ethnically-diverse population. Diabetic Medicine. 2009;26**(1)**:117-8.
16. Lorber D. Preconception Management for the Woman with Diabetes. Diabetes Spectrum. 1995; 8(5):269-300.
17. Matsuba I. [Life guidance and family planning for diabetic patient and family members]. Nihon rinsho Japanese journal of clinical medicine. 1997;55 Suppl:402-7.
18. Matsuba I, Ikeda Y. [Life guidance and family planning for diabetic patient and family members]. Nihon rinsho Japanese journal of clinical medicine. 1990;48 Suppl:842-7.
19. MD EK. PCCfor Women with Type 1 and Type 2 Diabetes—The Same But Different Canadian Journal of Diabetes. 2012; 36**(2)**:4.
20. Moloney YS, John O'Hare, James Saunders, Jean. . Pre-conception planned pregnancy improves glycaemic control in women with diabetes. Journal of Diabetes Nursing. 2011.
21. Norgaard SK, Nichum VL, Barfred C, Juul HM, Secher AL, Ringholm L, et al. Use of the smartphone application "Pregnant with Diabetes". Danish medical journal. 2017;64**(11)**.
22. Posner SF, Broussard DL, Sappenfield WM, Streeter N, Zapata LB, Peck MG. Where are the data to drive policy changes for preconception health and health care? Women's health issues: official publication of the Jacobs Institute of Women's Health. 2008;18**(6):**S81-6.
23. Posner SF, Johnson K, Parker C, Atrash H, Biermann J. The national summit on preconception care: a summary of concepts and recommendations. Matern Child Health J. 2006;10**(5)**:S197-205.
24. Schmiegelow MD, Andersson C, Kober L, Andersen SS, Norgaard ML, Jensen TB, et al. Associations between body mass index and development of metabolic disorders in fertile women--a nationwide cohort study. J Am Heart Assoc. 2014;3(2):e000672.
25. Schneider S, Freerksen N, Maul H, Roehrig S, Fischer B, Hoeft B. Risk groups and maternal-neonatal complications of preeclampsia--current results from the national German Perinatal Quality Registry. J Perinat Med. 2011;39**(3):**257-65.
26. Schneider S, Freerksen N, Rohrig S, Hoeft B, Maul H. Gestational diabetes and preeclampsia--similar risk factor profiles? Early Hum Dev. 2012;88**(3):**179-84.
27. Schneiderman EH. Gestational diabetes: an overview of a growing health concern for women. Journal of infusion nursing: the official publication of the Infusion Nurses Society. 2010;33**(1):**48-54.
28. Schneid-Kofman N, Sheiner E, Levy A, Holcberg G. Risk factors for wound infection following cesarean deliveries. International journal of gynaecology and obstetrics: the official organ of the International Federation of Gynaecology and Obstetrics. 2005;90(1):10-5.
29. Schoch JJ, Hunjan MK, Anderson KR, Lohse CM, Hand JL, Davis DMR, et al. Temporal trends in prenatal risk factors for the development of infantile hemangiomas. Pediatric dermatology. 2018;35**(6)**:787-91.
30. Schoenaker D, Vergouwe Y, Soedamah-Muthu SS, Callaway LK, Mishra GD. Preconception risk of gestational diabetes: Development of a prediction model in nulliparous Australian women. Diabetes Res Clin Pract. 2018; 146:48-57.
31. Schoenaker DA, Soedamah-Muthu SS, Callaway LK, Mishra GD. Pre-pregnancy dietary patterns and risk of gestational diabetes mellitus: results from an Australian population-based prospective cohort study. Diabetologia. 2015;58**(12):**2726-35.
32. Schoenaker DA, Soedamah-Muthu SS, Mishra GD. Quantifying the mediating effect of body mass index on the relation between a Mediterranean diet and development of maternal pregnancy complications: the Australian Longitudinal Study on Women's Health. The American journal of clinical nutrition. 2016;104**(3)**:638-45.
33. Scholing JM, Olthof MR, Jonker FA, Vrijkotte TG. Association between pre-pregnancy weight status and maternal micronutrient status in early pregnancy. Public health nutrition. 2018;21**(11):**2046-55.
34. Schrander-Stumpel C. Preconception care: challenge of the new millennium? Am J Med Genet. 1999;89**(2)**:58-61.
35. Tripathi A, Rankin J, Aarvold J, Chandler C, Bell R. Preconception counseling in women with diabetes: a population-based study in the north of England. Diabetes Care. 2010;33**(3):**586-8.
36. Yamamoto JM, Hughes DJF, Evans ML, Karunakaran V, Clark JDA, Morrish NJ, et al. Community-based pre-pregnancy care programme improves pregnancy preparation in women with pregestational diabetes. Diabetologia. 2018;61**(7):**1528-37.
