# Supplementary material for: Systematic review and meta-analysis of the effectiveness of pre-pregnancy care for women with diabetes for improving maternal and perinatal outcomes
Source: PLoS One. 2020 Aug 18;15(8):e0237571. doi: 10.1371/journal.pone.0237571 (PMC7433888; doi:10.1371/journal.pone.0237571)
Supplement: S3 File — (DOCX) [file pone.0237571.s004.docx]

**Sensitivity analysis by restricting studies with high risk of bias as measured by the New Castel Ottwa Scale (NOS) for observational studies**


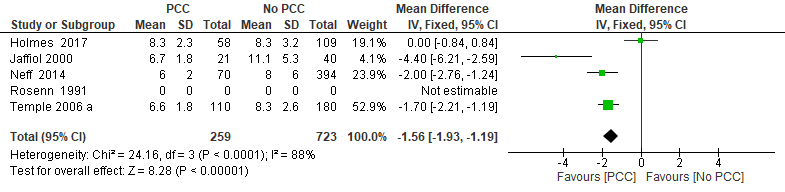


**Supplementary Fig 1. Mean gestational age at the time of the first antenatal visit from four studies of women with pre-existing diabetes mellitus who did or did not receive preconception care, one study with high risk of bias is not estimated.**

The black diamond represents the pooled difference estimate. Heterogeneity is quantified by I^2^ statistics, an I^2^ value ≥ 50 indicates substantial heterogeneity. Estimated results are presented as mean difference with 95% Confidence Interval. PCC= Preconception care; No PCC= No preconception care; CI= Confidence intervals.


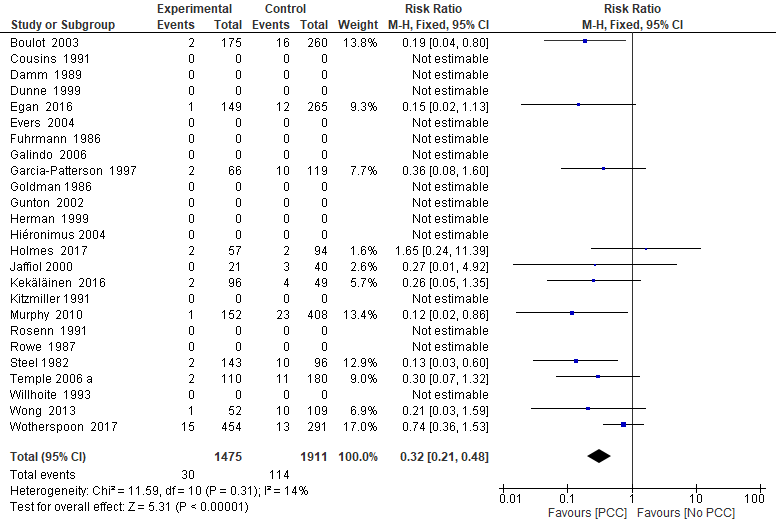


**Supplementary Fig 2. Risk ratio for congenital malformation from 11 studies of women with pre-existing diabetes mellitus who did or did not receive preconception care, 14 studies with high risk of bias are not estimated.**

The black diamond represents the pooled risk estimate. Heterogeneity is quantified by I^2^ statistics, an I^2^ value ≥ 50 indicates substantial heterogeneity. Estimated results are presented as risk ratio with 95% Confidence Interval. PCC= Preconception care; No PCC= No preconception care; CI= Confidence intervals.


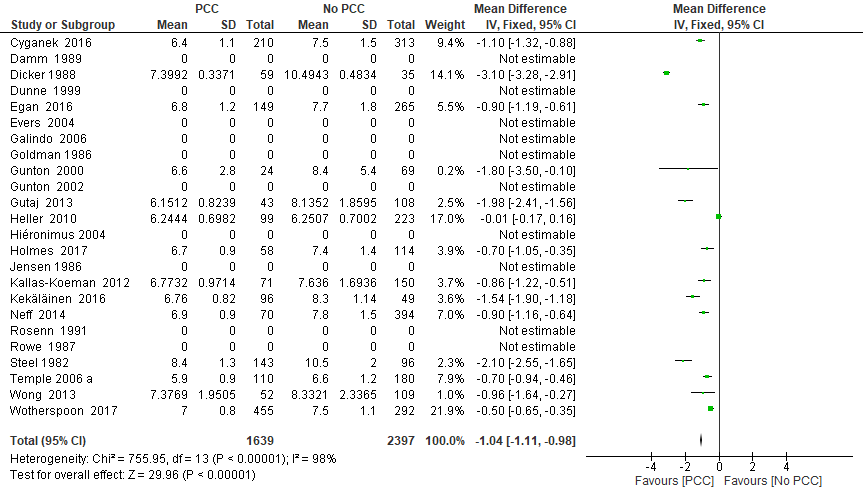


**Supplementary Fig 3. First trimester mean value of glycosylated haemoglobin (HbA1c) from 14 studies of women with pre-existing diabetes mellitus who did or did not receive preconception care, 10 studies with high risk of bias are not estimated.**

The black diamond represents the pooled difference estimate. Heterogeneity is quantified by I^2^ statistics, an I^2^ value ≥ 50 indicates substantial heterogeneity. Estimated results are presented as mean difference with 95% Confidence Interval. PCC= Preconception care; No PCC= No preconception care; CI= Confidence intervals.


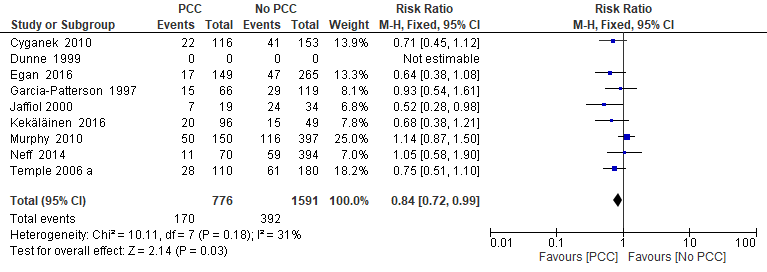


**Supplementary Fig 4. Risk ratio for preterm delivery from eight studies of women with pre-existing diabetes mellitus who did or did not receive preconception care, one study with high risk of bias is not estimated.**

The black diamond represents the pooled risk estimate. Heterogeneity is quantified by I^2^ statistics, an I^2^ value ≥ 50 indicates substantial heterogeneity. Estimated results are presented as risk ratio with 95% Confidence Interval. PCC= Preconception care; No PCC= No preconception care; CI= Confidence intervals.


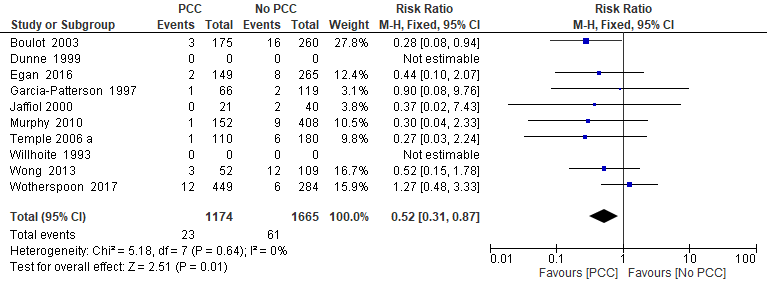


**Supplementary Fig 5. Risk ratio for perinatal mortality from eight studies of women with pre-existing diabetes mellitus who did or did not receive preconception care, two studies with high risk of bias are not estimated.**

The black diamond represents the pooled risk estimate. Heterogeneity is quantified by I^2^ statistics, an I^2^ value ≥ 50 indicates substantial heterogeneity. Estimated results are presented as risk ratio with 95% Confidence Interval. PCC= Preconception care; No PCC= No preconception care; CI= Confidence intervals.


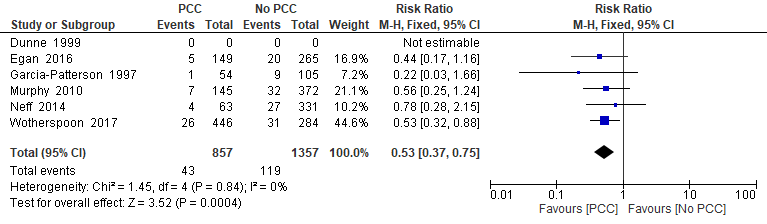


**Supplementary Fig 6. Risk ratio for small for gestational age from five studies of women with pre-existing diabetes mellitus who did or did not receive preconception care, one study with high risk of bias is not estimated.**

The black diamond represents the pooled risk estimate. Heterogeneity is quantified by I^2^ statistics, an I^2^ value ≥ 50 indicates substantial heterogeneity. Estimated results are presented as risk ratio with 95% Confidence Interval. PCC= Preconception care; No PCC= No preconception care; CI= Confidence intervals.


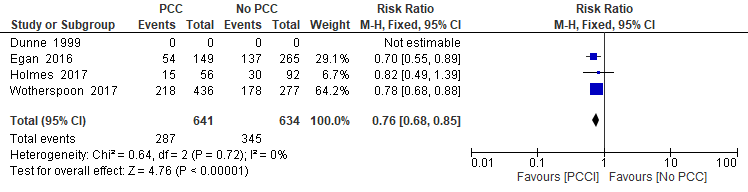


**Supplementary Fig 7. Risk ratio for neonatal intensive care admission from three studies of women with pre-existing diabetes mellitus who did or did not receive preconception care, one study with high risk of bias is not estimated.**

The black diamond represents the pooled risk estimate. Heterogeneity is quantified by I^2^ statistics, an I^2^ value ≥ 50 indicates substantial heterogeneity. Estimated results are presented as risk ratio with 95% Confidence Interval. PCC= Preconception care; No PCC= No preconception care; CI= Confidence intervals.


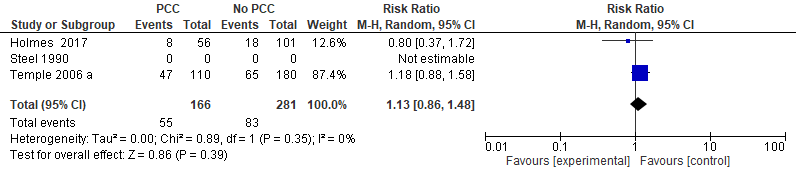


**Supplementary Fig 8. Risk ratio for maternal hypoglycemia from three studies of women with pre-existing diabetes mellitus who did or did not receive preconception care.**

Data of Steel 1990 were not estimated in the analysis. The large blue square represents the estimate effect of the study with he highest weight and very precise 95% CI. The black diamond represents the pooled risk estimate. Heterogeneity is quantified by I^2^ statistics, an I^2^ value ≥ 50 indicates substantial heterogeneity. Estimated results are presented as risk ratio with 95% Confidence Interval. PCC= Preconception care; No PCC= No preconception care; CI= Confidence intervals.
