# Supplementary material for: Systematic review and meta-analysis of the effectiveness of pre-pregnancy care for women with diabetes for improving maternal and perinatal outcomes
Source: PLoS One. 2020 Aug 18;15(8):e0237571. doi: 10.1371/journal.pone.0237571 (PMC7433888; doi:10.1371/journal.pone.0237571)
Supplement: S1 Fig — (DOCX) [file pone.0237571.s005.docx]

**Forest Plots**

**
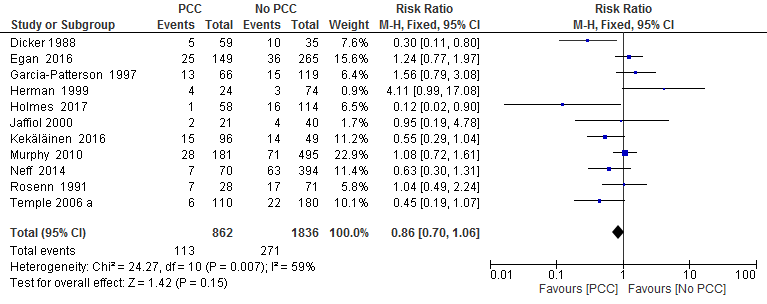
Figure 1. Risk ratio for miscarriage from 11 studies of women with pre-existing diabetes mellitus who did or did not receive preconception care**.

The black diamond represents the pooled risk estimate. Heterogeneity is quantified by I^2^ statistics, an I^2^ value ≥ 50 indicates substantial heterogeneity. Estimated results are presented as risk ratio with 95% Confidence Interval. PCC= Preconception care; No PCC= No preconception care; CI= Confidence intervals.

**Figure 2. Risk ratio for large for gestational age (LGA)/ macrosomia from nine studies of women with pre-**

**
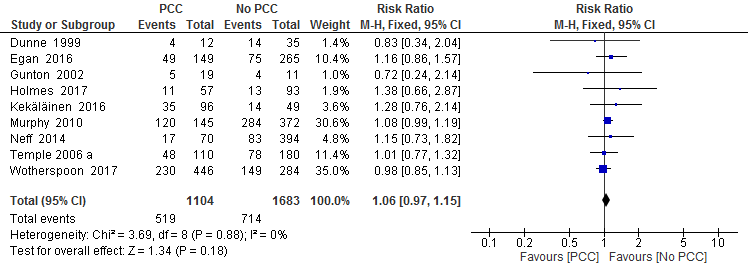
existing diabetes mellitus who did or did not receive preconception care.**

The black diamond represents the pooled risk estimate. Heterogeneity is quantified by I^2^ statistics, an I^2^ value ≥ 50 indicates substantial heterogeneity. Estimated results are presented as risk ratio with 95% Confidence Interval. PCC= Preconception care; No PCC= No preconception care; CI= Confidence intervals.


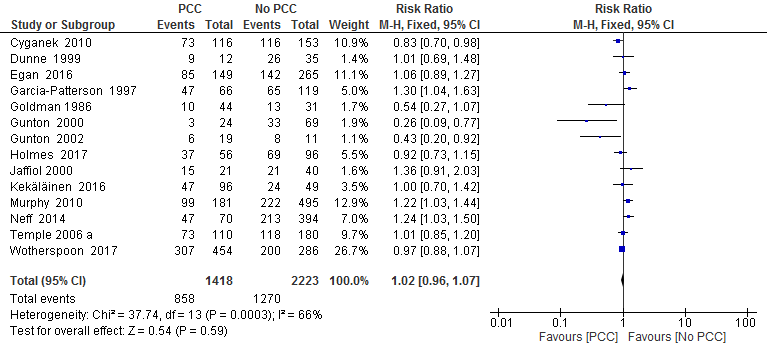
**Figure 3. Risk ratio for Caesarean section from 14 studies of women with pre-existing diabetes mellitus who did or did not receive preconception care.**

The black diamond represents the pooled risk estimate. Heterogeneity is quantified by I^2^ statistics, an I^2^ value ≥ 50 indicates substantial heterogeneity. Estimated results are presented as risk ratio with 95% Confidence Interval. PCC= Preconception care; No PCC= No preconception care; CI= Confidence intervals.

**Figure 4. Risk ratio for neonatal hypoglycaemia from five studies of women with pre-existing diabetes mellitus who did or did not receive preconception care.**

**
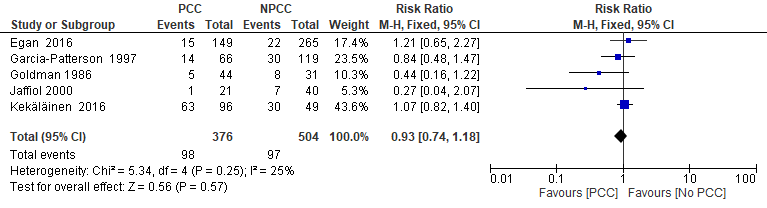
**

The black diamond represents the pooled risk estimate. Heterogeneity is quantified by I^2^ statistics, an I^2^ value ≥ 50 indicates substantial heterogeneity. Estimated results are presented as risk ratio with 95% Confidence Interval. PCC= Preconception care; No PCC= No preconception care; CI= Confidence intervals.

**
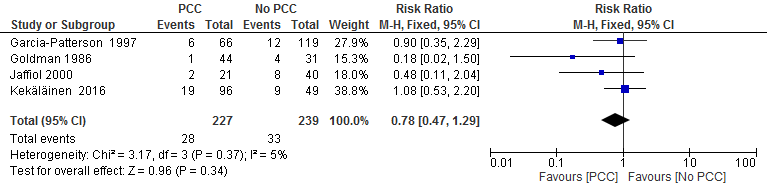
Figure 5. Risk ratio for neonatal respiratory distress from four studies of women with pre-existing diabetes mellitus who did or did not receive preconception care.**

The black diamond represents the pooled risk estimate. Heterogeneity is quantified by I^2^ statistics, an I^2^ value ≥ 50 indicates substantial heterogeneity. Estimated results are presented as risk ratio with 95% Confidence Interval. PCC= Preconception care; No PCC= No preconception care; CI= Confidence intervals.

**Figure 6. Risk ratio for shoulder dystocia from two studies of women with pre-existing diabetes mellitus who did or did not receive preconception care.**

**
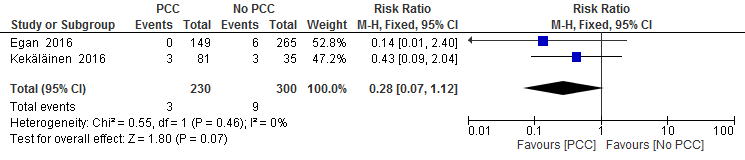
**The black diamond represents the pooled risk estimate. Heterogeneity is quantified by I^2^ statistics, an I^2^ value ≥ 50 indicates substantial heterogeneity. Estimated results are presented as risk ratio with 95% Confidence Interval. PCC= Preconception care; No PCC= No preconception care; CI= Confidence intervals.
