# Supplementary material for: Systematic review and meta-analysis of the effectiveness of pre-pregnancy care for women with diabetes for improving maternal and perinatal outcomes
Source: PLoS One. 2020 Aug 18;15(8):e0237571. doi: 10.1371/journal.pone.0237571 (PMC7433888; doi:10.1371/journal.pone.0237571)
Supplement: Attachment — Submitted filename: Reply to reviewers march 10.docx [file pone.0237571.s006.docx]

Dear Editors,

We would like to thank the reviewers for their informative and constructive comments, based on their directions, we considered their comments and revised all reported assessments numbers and figures.

Kindly find below our point-by-point reply for their comments:

Reviewer 1

We thank the reviewer for his/her meticulous revision of all the outcomes, in some instances we agreed with some of his/her comments and changed our assessment of the GRADE of evidence on certain outcomes. However, we need to explain that the GRADE tool is mainly used to give the policy makers and those who implement evidence some direction about the expected degree of success in improving the particular outcome if the intervention is implemented. Hence, the importance of the **effect size, heterogeneity, CI crossing no effect line** and the consistency of the **direction of outcomes** in all the studies and not only the methodological quality of evidence including bias. Another important consideration in our assessment is that all observational studies, **based on the GRADE approach (not our decision)**, start at a score of **(low)**, which is the third in a four-point scale assessing our certainty on the evidence

| Reviewer 1 comments | Action/comments |
| --- | --- |
| Title. I suggest that the same words are used in the title as in the manuscript i.e. pre-conception care. Not only complications in fetuses are studied but also complications in the newborn, maybe offspring or infants would be more appropriate words. | Reply: According to WHO, the **perinatal** period commences at 22 completed weeks (154 days) of gestation and ends seven completed days after birth which includes all the events reported in the current study. So we changed the term “fetal” to Perinatal” in the title. |
| Abstract.  I suggest that the results in the abstract should be re-evaluated, please see below my comments on the result in the main text. Could you also give the number of women with type 1 and type 2 diabetes included in the studies? | Reply: Some of the results have been modified according to the reviewer’s comments (please see reply to reviewer in results section). We could not find the exact number of women with type 1 and type II diabetes because some studies didn’t specify the numbers in each group, most probably because the effect of PCC does change with the type of diabetes unless there were vascular complications related to diabetes and those we have considered when we assessed the presence of confounders in our assessment of bias in the included studies (please see table 4).  . |
| The authors describe the importance of well-regulated glucose levels in pregnant women with diabetes, especially during the first trimester. Further, they state that management of diabetes is a challenge in low- and middle-income countries. Here the burden of diabetes is higher in the younger population thus increasing the risk of complications during pregnancy and in the fetus and newborn child. The variation of uptake of recommendations in different socioeconomic strata in all countries is also problematic. These are important issues, but the current study does not specifically address them as the review includes studies from high-income countries. The reasons for lack of knowledge of how to manage diabetes in low resource and socioeconomic under-privileged regions and what to do to amend this should be addressed in the discussion. | Reply: We included this information in the in introduction to portray a complete picture about the epidemiology od diabetes is a common health problem all over the world especially young age group and in different countries, hence, the importance of this review to introduce PCC as a preventive measure to most of the complications of diabetes in pregnancy. Further details were included in the discussion |
| **Methods:**  The description of search methods, study selection and identification, and data extraction are well described. Under subheading Quality Assessment the score using stars should be described. What does the stars stand for in the different domains? Describe this process in more detail. | Reply: the details of the tool (The Newcastle-Ottawa Scale), has been explained in details in the methods section with its cited reference. (page 8, lines 186-193) |
| In Table 4 for stars are used for bias assessment and their use should be explained as this assessment is crucial for the results of the meta-analyses. | Reply: the details of staring studies was explained in details in the methods section with its cited reference. (page 8, lines 186-193) |
| Reference #20 is wrong (line 193), it should be #21. | Corrected |
| Line 195; the word trials is misleading as the current literature review only comprise one trial, all other studies are observational. | Corrected to studies |
| Assessment of the quality of the evidence (page 9). This section should be thoroughly revised. The symbols of the GRADE tool (used in table 6) should be described and how the design of a study gives the study a default grade should be explained. For instance, all observational studies are not default of low quality of evidence. Well-performed cohort studies have usually moderate quality of evidence. Further, reasons for down and up-grading the quality should be summarized, preferably in a supplementary table. Up-grading could also be done if there is a dose-response effect not only if the effect of intervention is great. Albeit, upgrading should be used with care.  Some details: line 212 the table Summary of findings in the manuscript should be referred to. The statistical analysis is performed with statistical well-known tools and seems appropriate. | Reply: All the details of the GRADE approach are addressed clearly in the methods section according to reference 21.  Kindly find this reference for more explanation and clarifications of the GRADE approach. <https://www.jclinepi.com/article/S0895-4356(13)00057-7/fulltext>  Please refer to the explanations at the top of the reply to reviewers |
| The numbers of full text article is not the same in the text (n=75) and in Figure 1 (n=76) the same applies to excluded studies n=35 and n=36 respectively, please check and revise. | Revised and corrected |
| Also check the numbers in table 1. Two studies in the table are not mentioned in the text (20 and 31). | All studies mentioned in the table and text were revised and matched for all outcomes |
| Line 261: “..16 were retrospective studies..” but the number within brackets is 17 | All studies mentioned in the table and text were revised and matched for all outcomes |
| Under subheading Interventions the references in the text are not congruent with those in table 1 | All studies mentioned in the table and text were revised and matched for all outcomes |
| The same apply to the references in the text with subheading Outcome measures. | All studies mentioned in the table and text were revised and matched for all outcomes |
| In the table two connected studies are presented as one which is confusing. It would be easier for the reader to check the literature if the articles in the table are presented consecutively according to their reference number. | Unfortunately, this is nor possible because some authors (e,g. Temple ) has more than one publication in two different medical journals but are different outcomes for the same cohort. For the reader to refer to the certain study they have to find the correct citation. |
| Subheading “Effects of intervention”.  Lines 5-8. Gestational age at booking for antenatal care. The statement on quality of evidence is not supported by the data, albeit the effect of PCC is small. Five studies are not a small number of studies and the number of individuals included is large and the risk of bias as stated by the authors is moderate according to table 6, this should be congruent with the text. The number of studies, if more than one, is not a reason for down-grading of evidence. I suggest your conclusion of very low-quality of evidence should be reconsidered. | Reply: The quality of evidence was downgraded from low-grade (observational study) to very low -grade due to high- risk of bias in the study with the largest weight (Rossen 1991) and high unexplained heterogeneity (table 6). |
| Figure 2: the green symbol of reference Rosenn 1991 should be explained in the Table text (large drop-out, 50%). Usually studies with a drop-out over 20% are excluded from analysis. Reconsider if this study should be excluded from the analysis? The same applies for Rosenn in Figure 4. | Reply: In the protocol, we did not define drop-off of more than 20% to indicate study exclusion. Loss of follow up is appraised in the NOS for assessment of bias.  The green symbol is present in all the studies in this forest plot and it indicates the roughly the (mean). It is more prominent for the Rosenn 1999 because the small confidence interval (-1.39 to -1.21) which masked the arms of the confidence interval (the two ends of the black line). |
| Lines 12-17. Congenital malformation. One study should be excluded from the Forrest plot since it does not contribute to the result (Dunne 1999). | Reply: Dunne 1999 reported the congenital anomalies and they reported no cases of congenital anomalies in the intervention or the control groups as (ZERO) is a number and the number of participants should be included in the denominator of the pooled estimate we included the study in the forest Plot.  Kindly check this reference:  <https://bmcmedresmethodol.biomedcentral.com/articles/10.1186/1471-2288-7-5> |
| Also an analysis excluding studies with high risk for bias should be performed in order to investigate the impact on the outcome, see also Discussion, line 117-121. Please also check the congruence of numbers in text and table. | Reply: According to our protocol, we did not plan to do sensitivity analysis based on risk of bias as it is considered in the GRADE approach for quality of evidence. Additionally, kindly see below one example of the forest plot of HbA1c where we excluded all high biased studies (10 studies) however, the pooled effect of PCC on the reduction of HbA1c stayed nearly the same. |
| Line 21-28. HbA1c. The conclusion of high-quality of evidence is not supported by Table 6 where the outcome is designated moderate quality of evidence. | Reply: we considered the number of participants in studies with high risk of bias versus number in studies with low risk. We added the number of participants in explaining GRADE evaluation in each outcome. |
| Lines 32-35. Maternal hypoglycemia. All three studies in the meta-analysis are assessed by the authors having low risk of bias. Reconsider the conclusion of very low-quality of evidence. | Reply: Please consider that we start from low certainty because of observational studies. From the forest plot, there is no consistency (different direction of effect) , large CI (1.07-1.79) which is not precise and high heterogeneity all these factors affected our certainty of evidence to be very low from low. It means that we are uncertain if the PCC if implemented mothers will have hypoglycemia or not !!! |
| In Figure 5 the text on x-axis (experimental and control) should be replaced to PCC and non-PCC. | Reply: Corrected |
| Lines 39-42. Preterm delivery. The authors state that the outcome has moderate-quality of evidence due to small effect. The reason for small effect could be that this is actually the true outcome. Small effect size is not conferring down-grading of evidence as is the opposite (large effect could increase the quality of evidence). All included studies (except Dunne 1999) have low risk of bias (the most important weight for assessment of evidence as stated on page 34 lines 25-26 in the manuscript and I fully agree with that). The conclusion should be reconsidered. | Reply: We have actually upgraded the evidence from low to moderate due to the reasons you have mentioned in your comments, however we could not improve our certainty of the effect more because of the small effect size. The GRADE approach considers the implications to clinical practice which can be extracted from the body of evidence, e.g does the evidence support that if PCC is implemented one should expect that preterm delivery will be reduced? If the effect size is small then the certainty that will definitely result in reduction of pre-term delivery will be lower because other factors (e.g maternal age, parity, socioeconomic factors…) may play a bigger role in reduction of PTD. Hence the moderate quality of evidence    Please see up/downgrading of the quality of evidence is according to guidelines of GRADE.  <https://www.jclinepi.com/article/S0895-4356(13)00057-7/fulltext> |
| In the Discussion the global burden of preterm delivery is discussed, the limitation of this review is that the studies are from high-income countries. There is a lack of studies from under-privileged countries where PCC could have a greater impact on this outcome. | We have suggested more than one solution for the provision of PCC globally. Please refer to pp 41-42 lines 215-220 |
| Lines 46-50. Perinatal mortality. Similarly, to “preterm delivery”. The quality of evidence is not only affected by the effect size (here presumed to increase it) but relies on the quality of the included studies. The reduced risk of perinatal mortality is mainly driven by two older studies Boulot 2003 (low-risk of bias) and Willhoite 1993 (high risk of bias due to differences in baseline characteristics for instance duration of diabetes and difference in prenatal care in the two groups). I suggest that Willhoite and Dunne 1999 is excluded from analysis because of the high risk of bias for those studies. The data should be re-analyzed. | Reply: According to our protocol which was published in the, we did not plan to do sensitivity analysis based on risk of bias, based on the fact that risk of bias of the whole body of evidence (all nine studies) is one of five factors considered in the GRADE approach for quality of evidence. Please refer to the above replies for assessment of GRADE OF EVIDENCE  (Downgrading of the quality of evidence is according to guidelines of GRADE.  <https://www.jclinepi.com/article/S0895-4356(13)00057-7/fulltext> |
| Lines 56-58. Small for gestational age. The included 6 studies have all (except Dunne 1999) low risk of bias and a large number of participants. Reconsider the conclusion of low quality evidence according to my comments to the preceding analyses of preterm delivery and SGA.  Lines 62-65. NICU admission. Low risk of bias for three studies including a large number of participant. Reconsider the conclusion. | Reply: Agree, reconsidered please see text |
| Line 69-75. Other outcomes. Many of these outcomes are based on studies with low risk of bias (only shoulder dystocia has low precision (due to low number of events). Some analyses point towards no effect of PCC on outcome with a high quality of evidence. Reconsider the results. | Reply: Please look at the CIs of all these outcomes which crossed the no effect line (which is 1), which means it may be effective and it may not be effective (irrespective of all other factors of upgrading or down grading of evidence). In addition, all observational studies started from (low) GRADE and the reasons for upgrading have been mentioned in our methodology in p 9, lines 218-223 |
| Publication bias. I don’t understand how heterogeneity in outcome can explain publication bias in reporting of HbA1c. Can you explain this further? | Reply: Heterogeneity does not give publication bias but when there is marked heterogeneity the funnel plot gives a distribution of the studies similar to publication bias.  Kindly refer to reference 69 Terrine 2005 for further explanation |
| Can you present data on the number of patients with type 1 and type 2 diabetes in the studies? Were there differences in outcome of PCC in the two conditions? | type 1 and type II patients were reported collectively as some studies did not report the outcomes of each group separately. |
| **Discussion.**  The authors state that PCC has an impact on several outcomes concerning the mother and the off-spring. The impact might be less or even non-existent in other outcomes should also be reported. The evidence for no difference between intervention and no intervention could be of sufficient quality (for instance if large studies were included in the analysis). This might be very valuable knowledge for maternity health care planners. | Reply: All the outcomes were summarized in the first paragraph and then most of them were elaborated furthermore in the discussion. Other outcomes were discussed on P 40 lines 167-172 |
| Line 117. The authors state that the high quality of evidence on PCC and congenital malformation is mainly based on the large effect size. The upgrading of evidence due to effect size should be used with great caution. The high risk of bias in nearly half of the studies should also be a caveat making the meta-analysis more uncertain, see also my comments to this outcome in the Result section. Still, moderate quality of evidence could be good enough for introduction of an intervention and a greater size of effect have a role for this decision. | Reply: revised and explained please refer to the text |
| Line 122-136. One reason for the lower risk of congenital malformation is better control of blood glucose during pregnancy. Other studies (78-81) have shown a dose-response effect of HbA1c level on congenital malformations, did the authors find dose-response effects in the current study? If this was the case an upgrading of the quality of evidence could be considered. | Reply: these data are not available in the included study to perform this analysis. |
| The authors point to a very important issue in studies with a great time span. Practice changes over time (i.e. different criteria for diabetes diagnosis, and introduction of continuous glucose measurements) and this could be a reason for excluding older studies or perform analyses of studies from different time intervals. A more comprehensive care of women in fertile ages and in pregnant women with diabetes could also diminish the positive effect of specific PCC programs. The effect of PCC could also vary in areas with different socioeconomic status and between high, middle and low income countries which also the authors state in lines 196-198. This issues could be further elaborated in the sections of implications for practice and research, lines 231-246 and joined with the text from lines 208-211. | Reply: in the protocol, sensitivity analysis was planned for unexplained high heterogeneity only, not for bias or temporal reasons |
| Lines 157-169. I don’t agree that there were a small number of studies supporting the effect of PCC on SGA. See my comments in the Result section. | Reply: Corrected |
| Line 179-181. Can you explain what you mean with the true effect and how it would be substantially different? In what way? See also my comments to this outcome in the Result section. | Reply: As a sequence of the uncertainty of the available evidence, the true effect can be different from the reported one either in direction or magnitude or both. |
| The result of this review is rather clear. PCC increases the health of mothers with diabetes and their off-spring in high-income countries. A limitation that leaves gaps of knowledge is that studies from middle and low-income countries were lacking. This is especially important as the authors focus on these issues in the Introduction of the manuscript.  The authors describe some studies using surveys and qualitative design to investigate women’s attitudes towards pregnancy planning in Britain and the US and one study of risk factors for SGA in Brazil. The results from these studies might help formulate strategies to reach women with diabetes in childbearing age at risk, in underprivileged areas, and in middle and low-income countries. | We had to discuss this issue in the discussion and not in the introduction because it became apparent after we have done the review  Indeed these studies will be helpful especially if they collectively analyzed in a qualitative review. However we cannot comment on that because we don’t know how many studies are available and what their quality are |
| References.  The references should be scrutinized for typos and incompleteness. For instance, #20, #23, #47, #81. | Reply: corrected |
| General remarks.  A linguistic revision of the text is needed and there are many typos and lack of spaces in the text. | Reply: Corrected |

Reviewer 2

We thank the reviewer for his/her meticulous review of the number included in the review which was of great help to us

| Reviewer 2 comments | Action/comments |
| --- | --- |
| Trying to calculate some figures, I find the total number of participants in the cohort studies to be 8361, in the article given as 8324 (p. 10, l. 262). | Recalculation: PCC:3213, NOPCC: 4986. Total: 8199   - In Gunton(2000): reported a total of 61 women.   24 pregnancies were reported in PCC (some participants had more than one pregnancy) and 69 pregnancies were reported in NO-PCC (some participants had more than one pregnancy)   - Gunton (2002): reported a total of 31 women. 19 pregnancies were reported in PCC (some participants had more than one pregnancy) and 16 pregnancies were reported (some participants had more than one pregnancy - Kallas-Koeman (2012): Data available for only 71and 150 participants. |
| Data for some participants are missing. I found data from 8040 participants possible for meta-analysis, in the paper the number is 8026 (p. 34, l. 3). | Corrected:  8199 participants |
| I calculated the number of subjects studied for HbA1c to be 4907, in the paper given as 4927. | Recalculated: the correct number is 4927, please see first forest plots which is matching the number in table 1 and in the text |
| Have I misunderstood some details on pp. 34-37 (effects of intervention)? | Please note that some forest plots are shown in supplementary files (only 8 in the manuscript), all outcomes are listed in the text. Summary of findings table includes only the important outcomes. |
| Congenital malformations - text: 24 studies, 5856 women, Table 5: 25 studies, 5903 women | Revised and Corrected:  : the total number of included studies is 25 with a total of 5903 participants. The number corrected in the text. |
| Perinatal mortality - text: ten studies, 3071 women, Table 5: 9 studies, 3024 women | Revised and corrected:  10 studies with 3071 women |
| p. 8, l. 189 and l. 190 - should probably read "low risk of bias" and "high risk of bias", respectively, instead of parentheses. Also: - at least one STAR instead of start. | Revised and corrected: |
| p.8, l. 204 - represents the SLANDERED error ... should probably read STANDARD error? | Revised and corrected: |
| p. 9, l. 225 - we assessED the quality ... | Revised and corrected: |
| p. 11, l. 264 - and 180 women in the trial, going through 270 pregnancies (it seems that the calculations involve pregnancies and not women?) | Revised and corrected: |
| p. 17, regarding study 14/Gunton (45) What does "Total N= of women: 61" mean? | Revised and corrected:   - The numbers rewritten and explained in table 1 as following - In Gunton(2000):   24 pregnancies were reported in PCC (some participants had more than one pregnancy) and 69 pregnancies were reported in NO PCC (some participants had more than one pregnancy) |
| and regarding study 15/Gunton (45) - "Total Number of women:31"? | Revised and corrected:   - The numbers rewritten and explained in table 1 as following - Gunton (2002): reported as Total N= of women: 31 in table 1. 19 pregnancies were reported in PCC (some participants had more than one pregnancy) and 16 pregnancies were reported (some participants had more than one pregnancy |
| p. 40, l. 101 - slightly earliER booking | Revised and corrected: |
| l. 121 - participants were FROM studies ... | Revised and corrected: |
| p. 41, l. 131 -27% of participants were FROM studies ... | Revised and corrected: |
| l. 154 - were FROM one study ... | Revised and corrected: |
| p. 42, l. 168 - participants were FROM one study ... | Revised and corrected: |
| l. 177 - the participants were FROM two studies ... | Revised and corrected: |
| p. 43, L. 197 - LMICs should probably be defined, although many readers would know the abbreviation (Low and Middle Income Countries?) Regarding health economics, the different costs might all be given in the same currency, for comparison. | Revised and corrected: |
