# Supplementary material for: Systematic review and meta-analysis of the effectiveness of pre-pregnancy care for women with diabetes for improving maternal and perinatal outcomes
Source: PLoS One. 2020 Aug 18;15(8):e0237571. doi: 10.1371/journal.pone.0237571 (PMC7433888; doi:10.1371/journal.pone.0237571)
Supplement: Attachment — Submitted filename: Plos One reply to reviewers revision 2 final.docx [file pone.0237571.s007.docx]

Dear Editor and reviewers,

We would like to thank our reviewers for their constructive comments. We agreed with our reviewers on their feedback and all required changes were done accordingly.

Point-by-point reply:

**Reviewer #1: Thank you very much for your appreciation of my work with your manuscript.**

Dr Amel Fayed (corresponding author) and co-workers have answered to my review and done a lot of changes that are satisfying and thus increased the clarity of the manuscript. Still there are some issues that need to be addressed.
General.
I don’t think that we disagree on the great importance that evidence or lack of evidence for effect or lack of effect of different interventions are studied using systematic reviews and meta-analyses. It is crucial to guide health care staff as well other stakeholder in decisions of interventions demanding much resources. Before GRADING the evidence of different outcomes, the included studied are assessed for relevance and quality as you have described. In a conservative assessment, which I propose, studies with high drop-out rate (>30%) and low quality are excluded as the results of these studies are not possible to evaluate. They could be included but then the effect on the outcome must be assessed as the authors have done in presenting the percentage of studies of high risk of bias in the analysis. Observational studies can have very low-grade score, low GRADE score but some high-quality cohort studies can have moderate GRADE score. Thus, the GRADE score does not only depend on the design of the studies but also of their inherent quality. This is very important to recognize in issues that can´t be studied with randomized trials for ethical reasons as in the current systematic review.

Reply: We totally agree with the reviewer and we have conducted sensitivity analysis excluding high risk of bias studies from the analysis to confirm the statement. Please see page (35,36, supplement 4)

**Title**.

- Perinatal is an appropriate word, with lower case p. The word fetal is still used in the manuscript and should be changed for instance in line 135, 314. There might be more, so please scrutinize the manuscript.

Reply: Agree and Done

**Abstract.**

Conclusion: I suggest you use the term pre-gestational diabetes as there was no possibility to present the frequencies of typ 1 and type 2, respectively. The assessment of risk of hypoglycemia during first trimester was based on studies of type 1 diabetes, which ought to be pointed out in the manuscript. This lack of information in the rest of literature should be addressed in the manuscript for instance in the section on page 10 under the heading: Participants (line 266).

I can´t see any description of confounders such as other cardiovascular conditions (hypertension, lipid disturbances and obesity) in Table 4 as referred to by the authors.

Reply: Agree done as below

- Pregestational diabetes was used,
- (all reported results among type 1 diabetes) was added in the assessment of maternal hypoglycemia (page 12, line 310-311)
- “Most of studies did not report the differences in the outcomes among type 1 versus type 2 diabetes, as a result, we could not conduct the analysis separately for each type of diabetes” added to participants section, page 11, lines 285,286

**Introduction.**
The authors describe the variation of uptake of recommendations in different socioeconomic strata. In case “Low resource countries” are equivalent to “low income countries” use the latter term throughout the manuscript. If not define what you mean by low resource country. These are important issues the in the Discussion the authors should elaborate what to do to amend this. As there is a lack of studies addressing these problems it have implications for future research both quantitative and qualitative and this should be pointed out under subheading “Implications to research” (line 251).

Reply: Reply: Agree done as below

- “Low income countries” was used instead of low resource countries.
- There is lack of studies addressing these problems in the low-income countries that raise the need for future research both quantitative and qualitative, added to implication to research section, page 45, lines 244-246

**Methods.**
Under subheading Quality Assessment the score using stars is still not described neither in the text nor in the text accompanying Table 4. Further, assessment of the quality of the evidence and the symbols of the GRADE tool should be described, preferably in a supplementary table. I don’t think it is enough to refer to other publications.

Reply: Reply: Agree done as below

- This paragraph was added to the methods section (page 8, lines 224-250)

“The criteria assessed were: participants’ selection, comparability of groups and assessment of outcome for cohort studies. Participants’ selection, comparability of groups, and exposure criteria were used to assess the case-control studies. The maximum number of stars is nine: four stars awarded for selection-selection of exposed and non- exposed, ascertainment of exposure and temporal relation between exposure and outcome-, two for comparability if analysis controlled for confounder- and three stars awarded for outcome if the length of follow up was adequate, no attrition bias, and outcome was assessed independent of exposure Studies at “high risk of bias” score less than six stars or scores no stars in comparability domain irrespective of the number of stars scored.”

- This caption was added to table 4:

“Risk of bias was assessed using the Newcastle-Ottawa Scale (NOS). The number of stars represents the risk of bias, the maximum number of stars is nine, studies were classified as “low risk of bias” if they received a score of six stars or more and there is at least one star in the comparability domain. Studies at “high risk of bias” score less than six stars or scores no stars in comparability domain irrespective of the number of stars scored”

- This caption was added to the GRADE table

a Upgraded to high because of large effect size, consistency of direction of effect, no indirectness of evidence, and no heterogeneity or publication bias.

b Upgraded to moderate due to the narrow confidence intervals, consistency of direction of effect, no indirectness of evidence, and low risk of bias, no heterogeneity or publication bias.

c Downgraded to very low-grade due to the high risk of bias in the study with the largest weight (Rosenn 1991) and high unexplained heterogeneity

d Upgraded to moderate-certainty level because of low bias (77% of the participants were from studies at low risk of bias), while heterogeneity can be explained by long span of time between the first and the last study (1982 and 2017), The publication bias can be explained with the heterogeneity.

e Downgraded to very low-level certainty because of small number of studies, low bias, explained heterogeneity

f Upgraded to moderate-certainty level because of narrow confidence intervals, consistency of direction of effect, no indirectness of evidence, low risk of bias, low heterogeneity, no evidence of selective reporting.

G Upgraded to moderate-certainty level because the large effect size with precise narrow confidence interval, consistency of direction of effect, no indirectness of evidence, and no heterogeneity and no evidence of selective reporting.

**Results.**
The two connected studies (e.g. Temple) presented as one as they describe different outcomes in the same populations ought to be presented separately as the result are different outcomes if the authors want to make it easier for the reader to check the literature. The same applies if the articles in the table are presented consecutively according to their reference number.

Reply: Done

References are cited in order at first mention in the table of included studies, studies are ordered in an alphabetical order to make it easier to list. Results are reported from different studies separately as in (Temple a,b) cited as two different references.
Subheading “Effects of intervention”. However, it is not possible to reference them as one article as they were published in separate journals.

Gestational age at booking for antenatal care. I am satisfied that you omitted the statement of small number of studies. All symbol or acronyms used in a table (or a figure) such as the green dot should be explained in the explaining text or legend, in this case: green dot, calculated mean.

Reply: Done

Captions for all figures were updated
Congenital malformation, I accept your explanation. Line 21 change “form” to “from”.

Reply: Done
Line 28 change “form” to “from”.

Reply: Done
Maternal hypoglycemia. All three studies in the meta-analysis are assessed by the authors as having low risk of bias which means that they are of high quality although they have low numbers of events. This could increase the risk of bias. More importantly, the oldest (Steel et al) was published 1990 with data from the nineteen seventies and eighties before the meticulous surveillance of b-glucose and new recombinant, ultra-rapid acting insulins were introduced in contrast to Holmes et al. published 2017 and Temple et al who used data from 1990 to 2002. The heterogeneity could thus be caused by secular trends in treatment and surveillance. The 2 modern studies (Holmes and Temple) show no significant difference between PCC and controls. This implicates that maternal hypoglycemia in modern management of type 1 diabetic women (at least in high income countries) is not influenced by PCC. If the authors still consider Holmes and Temple having low risk of bias, I think that the evidence for this outcome should be low-quality, not very-low quality. I would say that you are not totally uncertain if PPC confers hypoglycemia or not compared to non-PCC, but it might not matter, still more studies could alter the evidence.

Reply: Done, we agree with the reviewer that more studies are needed to improve certainty

I think your explanation of the result concerning HbA1c applies here. The explanation of few studies shall be omitted from the text. I presume that your statement of true effect means that using modern data and more research would show if the risk of hypoglycemia is increased or decreased using PCC in a time with continuous glucose measuring (CGM) for an increasing part of patients with type 1 diabetes. You have already mentioned this in implications for research.

Reply: agree
Preterm delivery. Omit the sentence “The grade of evidence is considered moderate due to the small effect”. A small effect could be the true outcome. Adjust sentence in lines 52-53 …selective reporting increase our confidence in the outcome of a small reduction (4%) in preterm delivery.

Reply: Done

The sentence was omitted and the second sentence was adjusted.
Perinatal mortality. The upgrading is a too high. A reduction of RR to < 0.5 as in the current meta-analysis increase the grade by one star to moderate quality of evidence. RR < 0.2, which is not the case here, gives rise to two stars. The quality of evidence should thus be moderate.

Reply: Corrected in abstract, results and discussion
Small for gestational age. The RR is 0.52 not reaching the level <0.50 for upgrading one star. Thus up-grading is not an obvious action. If most included studies have low risk of bias the default grade score could be moderate quality. Upgrading to high is not appropriate here.

Reply: Corrected in abstract, results and discussion.
Other outcomes. Many of these outcomes are based on studies with low risk of bias. As the authors point out in the reply the CI of most studies cross the line depicting no significant difference between the PCC and non-PCC groups, which means that PCC might have no effect on these outcomes. Many studies with low bias and no difference between groups could render an upgrading, but I can accept your statement of low-certainty evidence.

Reply: Agree
**Discussion.**
The impact of PCC might be less or even non-existent in some outcomes. This should be summarized in the first paragraph as it is valuable knowledge for maternity health care planners. The risk for hypoglycemia should be elaborated further as one of the included studies describes management no longer used in high income countries.
Lines 142- 143. Alter to … “moderate quality albeit the effect was size was small”.
The authors point to a very important issue in studies with a great time span. Practice changes over time (i.e. different criteria for diabetes diagnosis, and introduction of continuous glucose measurements) this is especially important for the risk of hypoglycemia during pregnancy, which was much discussed earlier when stricter management began. I persists in the suggestion that you address this, even if you do no sensitivity analysis as this an important outcome that might need further studies with modern treatment. This should be addressed in connection to the last paragraph on page 40, lines 193-195. See also my comments in the result section.

Reply: Done

This paragraph was added to the discussion section

Meta-analysis results showed that the effect of PCC on maternal hypoglycemia was of low-certainty level. The three included studies had a low risk of bias but a high level of heterogeneity. Studies included were conducted in different time periods, during which tremendous changes were made with the management of diabetes, which explains the heterogeneity level. Evidence could be altered if more studies were to be carried out.

Sensitivity analysis was added according to level of quality of included studies (supplementary 4)

The result of this review is rather clear. PCC increases the health of mothers with diabetes and perinatal period of the off-spring in high-income countries. The authors describe some studies using surveys and qualitative design to investigate women’s attitudes towards pregnancy planning in Britain and the US and one study of risk factors for SGA in Brazil. The results from these studies might help formulate strategies to reach women with diabetes in childbearing age at risk, in underprivileged areas, and in low-income countries. This could be addressed in the section implications for research.

Reply: Done

This paragraph was added to implication to research

There is lack of studies addressing these problems in the low-income countries that raise the need for future research both quantitative and qualitative.
General remarks.
A further linguistic revision of the text is needed and there are still typos in the text.

Reply: Done

***Reviewer #2: With the large amount of data, the authors should check carefully the numbers in text and tables.***

**Abstract**
The effect on maternal hypoglycemia is reported to be RR 1,42;95% CI: 0,72-2,82 (p 2, l 60) and also in the Effects of intervention (p 32, l 36), while in Tables 5 and 6 it is said to be RR 1,38; 95% CI 1,07-1,79.

Reply: Corrected according to the tables

**Search methods**
..all the literature published up to March 2019 ..
This sentence might have included "between 1983 and .." - this information is given in Study Characteristics (p 10, l 268)., One study dated 1982 is also included, maybe this one was found linked to another study?

Reply: We mean studies from the commencement of each database till March 2019

Table 1
Cohort study 12 (Garcia-Patterson): a miscarriage rate of 13/66 gives 53 continuing pregnancies. The rate of SGA should probably read 1/53.
Similarly, the rate of RDS should probably read 12/119.

Reply: corrected

Table 2
Macrosomia rate in the NO-PCC group should probably read 4/12.

Reply: corrected
Assessment of the methodological quality of the included studies
Ref (40) is named Rosen et al (p 26, l 327). The correct spelling is Rosenn, as given in References, Tables 1 and 4.

Reply Corrected

Effects of intervention
Here the same reference is named Rossen (p 31, l 9).

Reply: Corrected

Gestational age at booking for antenatal care
MD 1,31(probably weeks? as it is translated into approximately ten days) - p 31, l 7.

Reply: Yes, it was computed as weeks in the mata-analysis

**Discussion**
Incremental increase ... (p 38, l 134) is superfluous.

Reply: Corrected to this sentence

“Many studies confirmed the incremental increase in the rate of adverse pregnancy outcomes with the increase in the level of HbA1c”
FORM should be corrected to FROM at several places (p 31, l 21, p 32, l 28 and 51, p 33, l 73)

Reply: Corrected

***Reviewer #3: Conduct a meta-analysis to evaluate the effectiveness and safety of pre-conception care in improving maternal and perinatal outcomes and evaluate the grade of the body of evidence for each outcome. They identify 36 studies and the meta-analysis results showed that PCC results in large reduction in congenital malformations, lowers HaA1c in the first trimester of pregnancy, lowers the preterm delivery rate***


1. **Abstract**: “the result… that PCC results in large reduction in congenital malformations, …owers HbA1c….” the causal effect was implied in the results. However, the study includes both trial and observational studies. The causal effect should be avoided throughout the manuscript.

Reply: revised.
2. Line 311. 21 studies were assigned to be at low risk of bias while 15 studies at high risk of bias. A sensitive analysis may be warranted to evaluate the robustness of the findings.

Reply: Done

- Sensitivity analysis according to the quality of included studies was done, all forest plots are added to Supplementary file 4, method of sensitivity analysis was added to the methods section and results of sensitivity analysis was added to the results section.

3. Figures 2 and 9. What’s the green rectangle in the figure 2 or blue rectangle in figure 9? Explanation to the figures are needed

Reply: explanation was added to all figures.
