# Supplementary material for: Systematic review and meta-analysis of the effectiveness of pre-pregnancy care for women with diabetes for improving maternal and perinatal outcomes
Source: PLoS One. 2020 Aug 18;15(8):e0237571. doi: 10.1371/journal.pone.0237571 (PMC7433888; doi:10.1371/journal.pone.0237571)
Supplement: Attachment — Submitted filename: reply to reviewers 3 july 22.docx [file pone.0237571.s008.docx]

**Dear Editor,**

**We would like to thank our reviewers for their constructive comments, and here are our point-by-point replies:**

**Title. Perinatal should be in lower case “perinatal”.**

Reply: Done
**Methods.
The caption of the GRADE table.
e. I don’t agree that the number of studies is low. Three studies can be good enough especially if they have low bias. The problem here is that the study with the greatest difference between intervention and control is very old and reflect a surveillance of blood glucose no longer in use. I suggest a wording such as “Downgraded to very low-level of certainty as there was a high heterogeneity due to major changes in surveillance of diabetes between the studies”.**

Reply: Corrected to high heterogeneity and inconsistency of direction of effect as the point estimate of each study is indifferent place in the forest plot
**Results.
I can´t see that the green dots in the figures are explained. Add an explanation in the captions of all illustrations.**

Reply: All graphs have explanation as the following

“The large green square represents the estimate effect of the study with the highest weight and very precise 95% CI. The black diamond represents the pooled difference estimate. Heterogeneity is quantified by I^2^ statistics, an I^2^ value ≥ 50 indicates substantial heterogeneity. Estimated results are presented as mean difference with 95% Confidence Interval. PCC= Preconception care; No PCC= No preconception care; CI= Confidence intervals.”

**Maternal hypoglycemia.
In table 6, Summary of findings, you state that PCC has no effect on hypoglycemia but in fact the RR is 1.38 (1.07-1.79) a significant difference. So the conclusion is that it seems to have an effect. You explain this finding in the Discussion in a satisfying way. In the text of the result section (page 33, line 36) I suggest that you alter the text to “PCC seems to have an effect on hypoglycemia during the first….) and omit small number of studies and emphasize the great span of time between studies one of which used blood glucose surveillance no longer in use.**

Reply: The paragraph was changed into the following paragraph

“We are uncertain about the effect of PCC on maternal hypoglycemia during the first trimester of pregnancy; (RR 1.38; 95% CI: 1.07- 1.79); three studies; 686 women; very low-certainty evidence) (Fig 5) (table 5). The grade of evidence was downgraded from low to very low due to inconsistency of the direction of effect and high heterogeneity (I2 =76%) in the included studies (table 6). The true effect is likely to be substantially different from the effect estimated in this review.”

**Discussion.
I can´t find the sensitivity analysis of maternal hypoglycemia that according to the authors is included in supplement 4**.

Reply : The three studies included in the assessment of hypoglycemia were all of low-risk of bias and the heterogeneity is explained by the long time span of the included studies, that is why we did not conduct sensitivity analysis for the maternal hypoglycemia outcome because we restricted the sensitivity analysis to outcomes with studies with high risk of bias as you have suggested. However, we decided to exclude the oldest study from the meta-analysis to support our discussion of its effect and not as sensitivity analysis, please see the Forest Plot below (S4 figure 8)

**Supplementary Fig 8. Risk ratio for maternal hypoglycemia from three studies of women with pre-existing diabetes mellitus who did or did not receive preconception care.**

Data of Steel 1990 were not estimated in the analysis. The large blue square represents the estimate effect of the study with he highest weight and very precise 95% CI. The black diamond represents the pooled risk estimate. Heterogeneity is quantified by I^2^ statistics, an I^2^ value ≥ 50 indicates substantial heterogeneity. Estimated results are presented as risk ratio with 95% Confidence Interval. PCC= Preconception care; No PCC= No preconception care; CI= Confidence intervals.

**There are still some typos in the manuscript. For instance, an extra dot in row 167, page 41.**

Reply: corrected

**In table 4 “Risk of bias assessment of the included studies (Steel 1982,1990) right column what is meant? The low risk due to age difference and number of smokers between the groups no regression??**

Reply: Corrected

There is no clinical difference between the studied groups regarding the age (27 vs 25), there is difference in other confounders such as number of smokers and no adjustment or regression analysis was done, so this study lost one stars in comparability and considered at “Low risk of bias”.

**In the text under Table 4 (page 31) Studies at “high risk of bias” score less than six stars or score no stars ….”**

Reply: Corrected to

Studies at “high risk of bias” scored less than six stars or scored no stars in the comparability domain, irrespective of the number of stars scored.
